# *n*-Iterative Exponential Forgetting Factor for EEG Signals Parameter Estimation

**DOI:** 10.1155/2018/4613740

**Published:** 2018-01-15

**Authors:** Karen Alicia Aguilar Cruz, María Teresa Zagaceta Álvarez, Rosaura Palma Orozco, José de Jesús Medel Juárez

**Affiliations:** ^1^Centro de Investigación en Computación, Instituto Politécnico Nacional (CIC-IPN), Avenida Juan de Dios Bátiz, Esq. Miguel Othón de Mendizábal, Col. Nueva Industrial Vallejo, Delegación Gustavo A. Madero, 07738 Ciudad de México, Mexico; ^2^Escuela Superior de Ingeniería Mecánica y Eléctrica, Unidad Azcapotzalco, Instituto Politécnico Nacional, Avenida de las Granjas, No. 682, Col. Santa Catarina, Delegación Azcapotzalco, 02250 Ciudad de México, Mexico; ^3^Escuela Superior de Cómputo, Instituto Politécnico Nacional, Avenida Juan de Dios Bátiz, Esq. Miguel Othón de Mendizábal, Col. Lindavista, Delegación Gustavo A. Madero, 07738 Ciudad de México, Mexico

## Abstract

Electroencephalograms (EEG) signals are of interest because of their relationship with physiological activities, allowing a description of motion, speaking, or thinking. Important research has been developed to take advantage of EEG using classification or predictor algorithms based on parameters that help to describe the signal behavior. Thus, great importance should be taken to feature extraction which is complicated for the Parameter Estimation (PE)–System Identification (SI) process. When based on an average approximation, nonstationary characteristics are presented. For PE the comparison of three forms of iterative-recursive uses of the Exponential Forgetting Factor (EFF) combined with a linear function to identify a synthetic stochastic signal is presented. The one with best results seen through the functional error is applied to approximate an EEG signal for a simple classification example, showing the effectiveness of our proposal.

## 1. Introduction

Electroencephalogram (EEG) is a technique to obtain information related to brain activity, extracting information measuring electric fields from the brain, allowing obtaining information related to the intention for different mental activities, like motor imagery, motor planning, imagined speech or subject identification [1]. When information from EEG is obtained their features should be processed and used in classification algorithms.

According to [2], neural signal oscillations are the most important EEG characteristics to study because the relationship among specific patterns, perceptual, motor, and emotional processes, is described by these changes. Because of EEG stochastic characteristics, an adaptive description is needed within the time analysis especially when changes are neither smooth nor slow [3, 4].

Nevertheless, their neural nature makes them difficult to analyze without using an adequate descriptor. Hence, new EEG signal modelling techniques allow selecting specific information helping neuropathology clinical studies [5] to obtain the parameters to be used in, for example, classification algorithms [6] such as Fuzzy Logic Classifier (FLC), Artificial Neural Networks (ANN), Particle Swarm Optimization, and Sliding Modes, [1, 7–10].

In [11] the use of Time-Frequency Distributions (TFD), Fast Fourier Transform (FFT), eigenvector methods (EM), Wavelet Transform (WT), and Auto-Regressive Method (ARM) is discussed, for EEG feature extraction in time and frequency domain. Results from this comparison indicate frequency methods may be not adequate for EEG signals while time-frequency do not give detailed information; now the election of one method will depend on the application objective [4, 5, 12].

The sampled neural signal in a mathematical sense corresponds to a stochastic, time variant, and nonlinear description with a specific bounded distribution function for each clinical case. From this, the neuron is represented by a Black Box (BB) system with only its excitation (input) and answer (output) available without knowing what happens inside.

Variations of Adaptive Auto-Regressive (AR) models have been proved to be adequate to model systems, where the number of parameters to determine depends on the model order. Other methods include Recursive Least Squares (RLS), Least Mean Squares (LMS), and Kalman Filter (KF) and their variations [9, 12–15]. In general algorithms by themselves are not adequate when abrupt changes are presented, giving rise to hybrid or correction forms such as Forgetting Factor (FF) [16]. The great importance of the identifier lies in describing the internal time system evolution and observing its stability and stationary properties [3, 9, 17].

**Figure 1:**
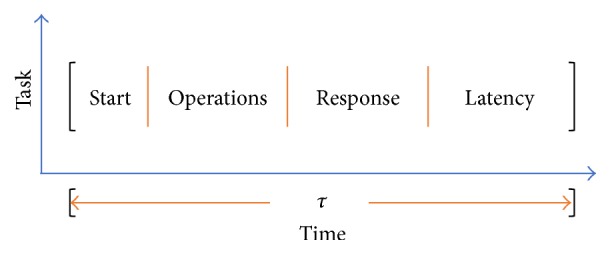
Representation of the processes for the corresponding time *τ*.

Considering computational operation latencies shown in Figure 1, the time interval between two consecutive output system steps makes the estimation-identification process achievable and feasible to add a second stage in the same interval, obtaining a recursive version using the Exponential Forgetting Factor (EFF) to modify the first identification.

A previous EFF analysis using (1) from [18] about the identification experiment was developed in [19], showing its effectiveness when using the sign function to give a correction factor, decreasing the identification error when implemented in nondeterministic signals:

(1) $$\begin{array}{c} EFF_{t}=sign\left(\;{\hat{A}}_{t}\;\right)e^{sign\left({\hat{A}}_{t}\right){\hat{e}}_{t}}, \end{array}$$

where ${\hat{A}}_{t}$ is the parameter estimated on average [20].

**Figure 2:**
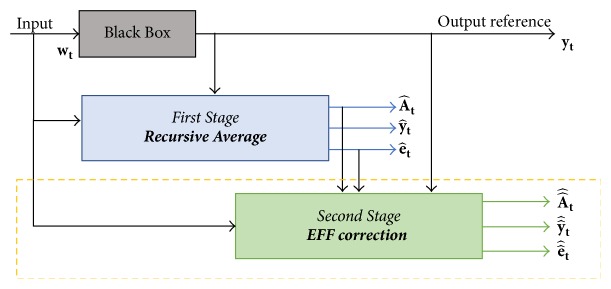
Block diagram for two stages into the estimation-identification process using a recursive average method (first) and the EFF (second, dashed line).

Equation (1) has been proved only for point to point corrections, integrating a second stage operation after the first developed on average, as shown in Figure 2.

Searching for a better identification, three different implementation cases of additional correction stages inside the second correction indicated in Figure 2 (dashed line) are compared. To accomplish this task, expression (1) is modified to create a recursive description for *y*_*t*_, based on stochastic input  *w*_*t*_, interacting in (2) and leading to the identification error ${\hat{e}}_{t}$ (3). Applying modifications in (1) the new parameter (4) is described and used instead of ${\hat{A}}_{t}$ in (2), obtaining a new identification and identified error.

(2) y^t=A^twt

(3) e^t=yt−y^t

(4) A^^t=A^t+EFFt−signA^t.

The obtained algorithms are proved using in the first-place sinusoidal signals and, then, the one with better results is applied to a synthetic amplitude and frequency variation.

## 2. Recursive Exponential Forgetting Factor (REFF) Comparison

**Figure 3:**
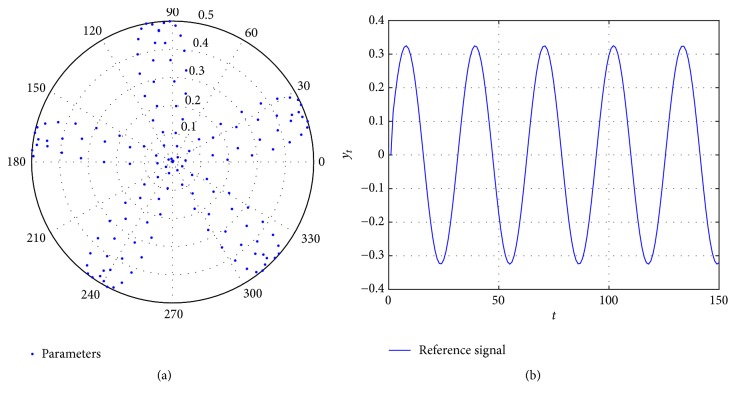
Reference parameters (a) and the output signal (b) to be identified.

For the following algorithms, their effectiveness is analyzed by comparing their corresponding recursive EFF parameters estimation and identifications with respect to the reference in Figure 3, whose sinusoidal shape (Figure 3(b)) is given by the parameters viewed in polar representation (Figure 3(a)).

The purpose of describing the parameters in a polar graph is to determine if they lead to instability problems due to their values. The identification is considered unstable and nonadequate when the estimation parameters go out of the unitary circle giving an overpass to the reference boundaries.

### 2.1. Recursion through the Previous Estimated Parameter

**Figure 4:**
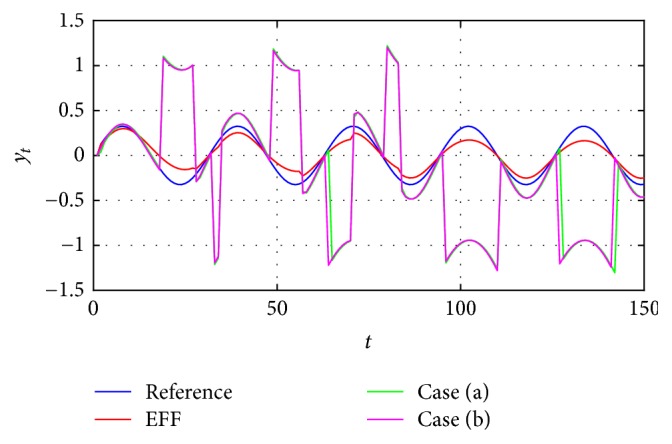
Identification using the estimation from case (a).

The first approach is by considering an instant *t* and the previous corrected estimated parameter ${\hat{\hat{A}}}_{t-1}$ based on the estimated parameter ${\hat{A}}_{t}$. For the second identification stage in (4) the estimation is described as ${\hat{\hat{A}}}_{t}$ in the iteration *t* as in (5). Results are shown in Figure 4.

(5) $$\begin{array}{c} {\hat{\hat{A}}}_{t}={\hat{\hat{A}}}_{t-1}+EFF_{t}-sign\left(\;{\hat{\hat{A}}}_{t-1}\;\right). \end{array}$$

### 2.2. Recursion through the Mean between the Previous and Actual Estimated Parameter

Continuing with (4) as the base, now the average estimated parameter for instant *t* and the delayed *t* − 1 corrected estimation as in (6) is considered. Results are shown in Figure 4.

(6) $$\begin{array}{c} {\hat{\hat{A}}}_{t}=\frac{{\hat{\hat{A}}}_{t-1}+{\hat{A}}_{t}}{2}+EFF_{t}-sign\left(\;\frac{{\hat{\hat{A}}}_{t-1}+{\hat{A}}_{t}}{2}\;\right). \end{array}$$

Notice in cases (a) and (b) a third stage is added to the process, having first the simple average approximation; secondly the EFF_*t*_ correction and the second parameters correction are made.

From Figure 4, it is possible to see that the identification using cases (a) and (b) is not adequate because every time the reference changes its concavity converging, it becomes more difficult, leading to undesirable peaks [15].

### 2.3. Iterative EFF-Estimation

In Figure 2 the dashed lines represent the first EFF correction; the same stage at the end of the process (7) is iteratively added, valid for *n* ≥ 2, having $a_{t,1}={\hat{A}}_{t}$ and $EFF_{t,1}=sign({\hat{A}}_{t})e^{sign({\hat{A}}_{t}){\hat{e}}_{t}}$ as the initial conditions, where *n* is the number of iterations made for the signal instant *t*, being *n* = 1 the average stage.

(7) $$\begin{array}{c} EFF_{t,n}=sign\left(\;a_{t,n}\;\right)e^{sign\left(a_{t,n}\right)e_{t,n}}, \end{array}$$

where *a*_*t*,*n*_ and *e*_*t*,*n*_ are defined as (8) and (9), respectively.

(8) at,n=at,n−1+EFFt,n−1−signat,n−1

(9) et,n=yt−yt,n.

Considering *y*_*t*,*n*_ as the identified signal after *n* iterations of the EFF and *y*_*t*_ as the output reference signal with input *w*_*t*_, then (9) could be defined as

(10) et,n=yt−at,nwt=yt−at,n−1+EFFt,n−1−signat,n−1wt.

**Figure 5:**
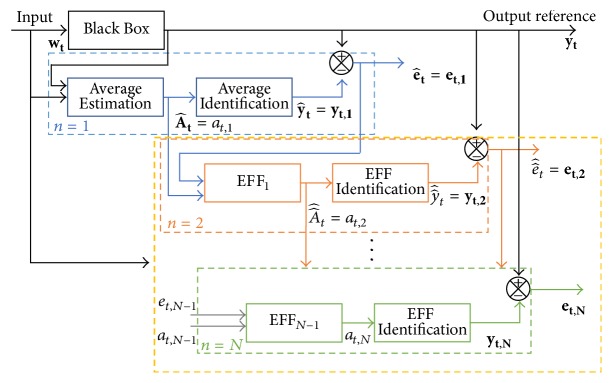
Block diagram for the estimation-identification process, case (c), with *n*-iterations of the EFF correction.

Unlike cases (a) and (b), this last one changes in estimating not only the parameter, but also the EFF, correcting *n* times the parameters to improve the identification signal convergence, as seen in the block diagram for case (c) shown in Figure 5.

The question now lies in how many iterations would be necessary to obtain an adequate convergence rate without having redundancies in data. The answer could vary according to what is most important from one application to another; thus, the reduction of the error is one of the main objectives in identification tasks.

**Figure 6:**
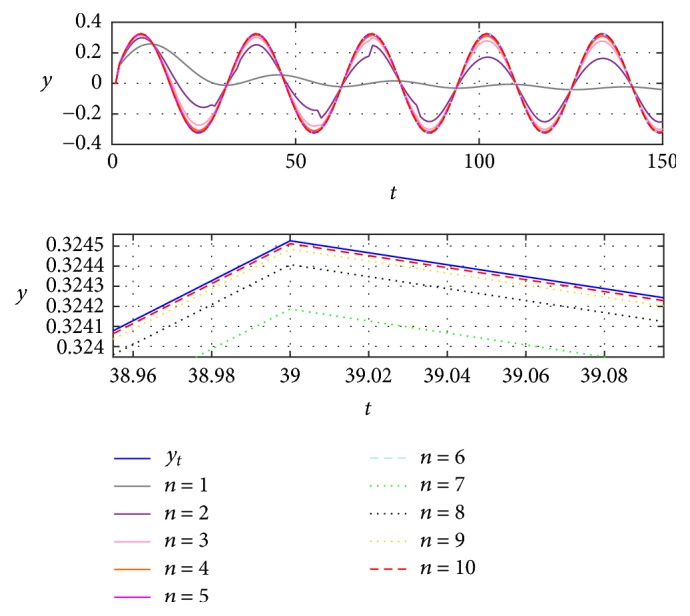
Identified signal applying case (c) for *n* = 10, from 2 to 10.

Thus, to verify the effectiveness of correction through the EFF, first the identified signals for *n* = 2 to *n* = 10 were obtained and ten simulations as shown in Figure 6.

**Figure 7:**
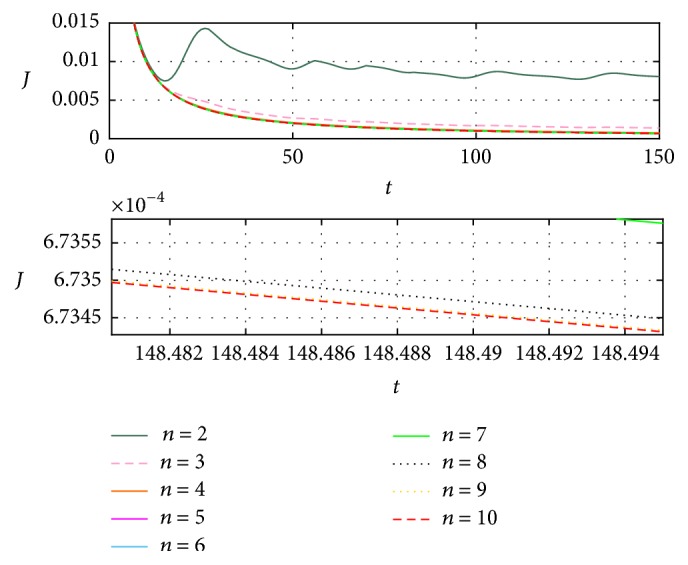
Comparison of the functional error *J*_*t*_ obtained from the identified signals from Figure 6.

Figure 6 shows that when increasing the *n* iterations, the identification is closer to the reference. Here is where the decision to increment the iterations depends on the desired accuracy level. To better appreciate the approximation errors, Figure 7 includes the functional error *J*_*t*_, in agreement with [21], with respect to previous closer identifications, as viewed in Figure 7.

**Figure 8:**
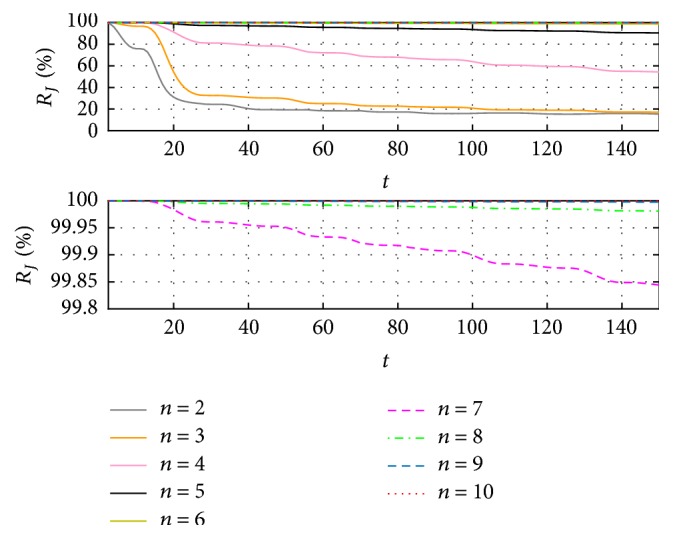
Resemblance errors and functionals, from consecutive EFF iterations.

The second process identifies a better convergence, as shown in Figure 7, where the error between the first and second iteration is remarkably reduced, as between the second and third, and so on. To have a better visualization from the differences between the results obtained in every iteration, Figure 8 shows the relation (11), expressed in percentage, which indicates how similar the results are in each iteration based on the functional error.

(11) $$\begin{array}{c} R_{J_{n}/J_{n-1}}=\frac{J_{t,n}}{J_{t,n-1}}\times 100\%. \end{array}$$

From iteration 7, the resemblance between it and the previous is above 99.8%, meaning the difference would be insignificant depending on the application. On the other hand, considering the magnitude errors obtained from this same iteration, they are less than 3% with respect to the original signal.

## 3. Parameter Estimation Example

**Figure 9:**
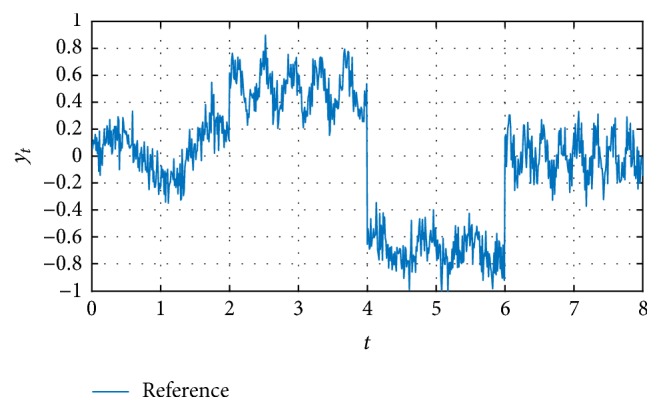
Reference signal described in (10), with evolution time *t* and sample frequency 100 Hz.

Our proposed example task is to approximate an EEG reference with changes in concavity and frequency with added noise, as those illustrated in [5, 22]. The reference signal is described as follows in (12), where *t* is the time evolution in seconds with sample frequency of 100 Hz, *f*_*θ*_ = 7, *f*_*β*_ = 25, *f*_*α*_ = 15, and *f*_*γ*_ = 40, as shown in Figure 9.

(12) $$\begin{array}{c} y_{t}=\left\{\begin{array}{cc} 2t^{0.5}\sin\left(2\pi f_{\theta }t\right) & t\in \left[0,2\right)\\ t^{0.5}\sin\left(2\pi f_{\beta }t\right) & t\in \left[0,4\right)\\ 2t^{0.25}\sin\left(2\pi f_{\alpha }t\right) & t\in \left[0,6\right)\\ 3t^{0.25}\cos\left(2\pi f_{\gamma }t\right) & t\in \left[0,8\right)\\ 0 & \text{otherwise.} \end{array}\right. \end{array}$$

**Figure 10:**
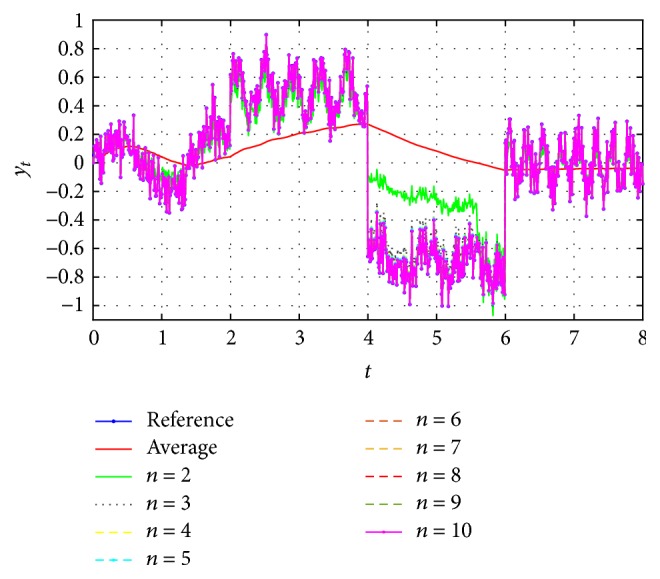
Identifications of the reference signal from Figure 9 applying an average identification and 10 iterations using recursive EFF.

**Figure 11:**
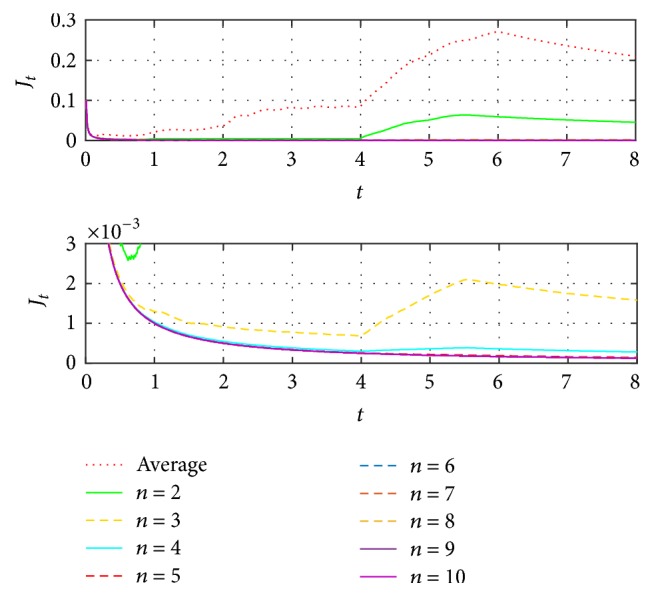
Functionals error obtained from the identification processes from Figure 10.

The estimation process generates the adequate parameters described symbolically as ${\hat{A}}_{t}$ in each sampled point for time *t*, approximating a linear variant function (2) to (12), which has stochastic properties, having variations in amplitude and frequency. The identification is composed of various iterations and the results are presented comparing the identifications with the proposed reference signal shown in Figures 10 and 11, observing the functional errors evolution.

**Figure 12:**
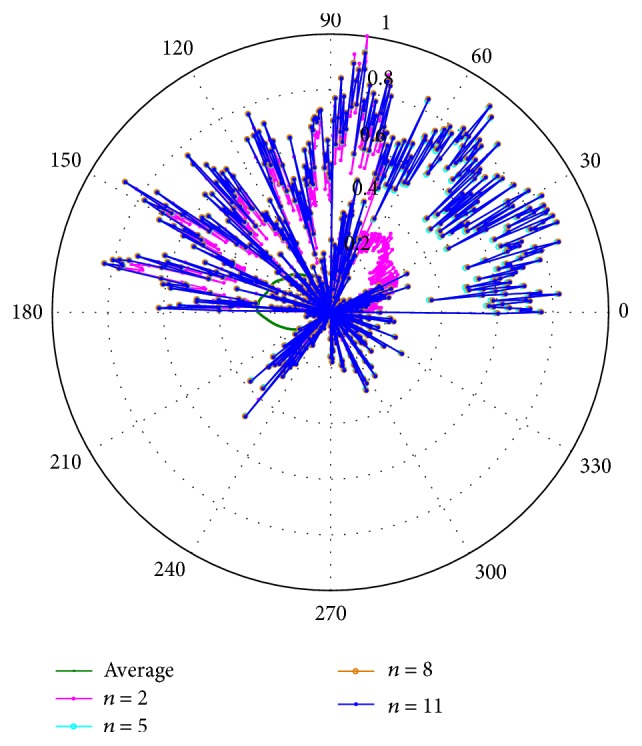
Parameters obtained using an average estimator (average), the simple EFF (*n* = 2), and 5, 8, and 11 iterations of the recursive description of the EFF.

In a Black Box (BB) system the relationship between the internal and external parameters cannot be made directly because the internal evolution is unknown, as would happen with real signals. Nevertheless, the parameters are important because they could be analyzed to obtain special features from EEG signals which are difficult to obtain for nonlinear signals. In fact, the obtainment of those parameters is the objective of the proposed technique.  Figure 12 presents the parameters obtained using the estimation, for the simple EFF and its iterative description considering *n* = 5, 8, and 11 iterations, which are representative when adding more than one correction step.

From Figure 12, it is determined that the parameters have variable characteristics for each sampled point. The variation between the average estimation and the simple EFF (*n* = 2) estimation is noticeably as in size and in direction. On the other hand, between the simple EFF and the other iterations the estimated parameters are similar in direction, presenting changes with the characteristic of never being able to leave the reference, maintaining the stability identification.

### 3.1. Estimation of EEG Signals

**Figure 13:**
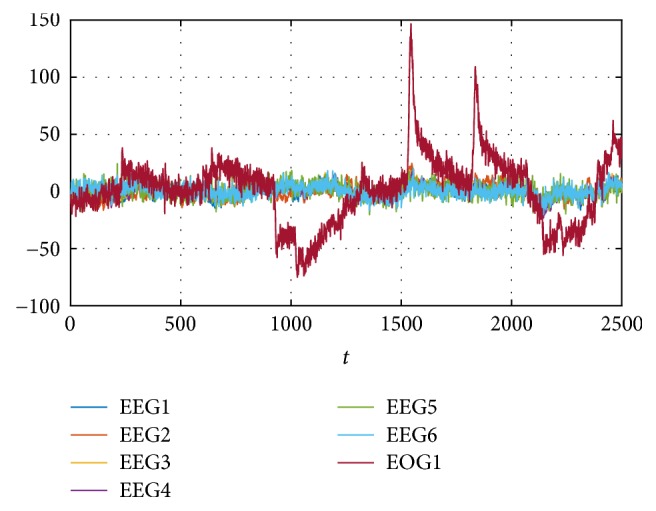
Sampled signals for Counting tasks, taken from [8, 9], for subject 1, considering 6 EEG channel and 1 EOG.

**Figure 14:**
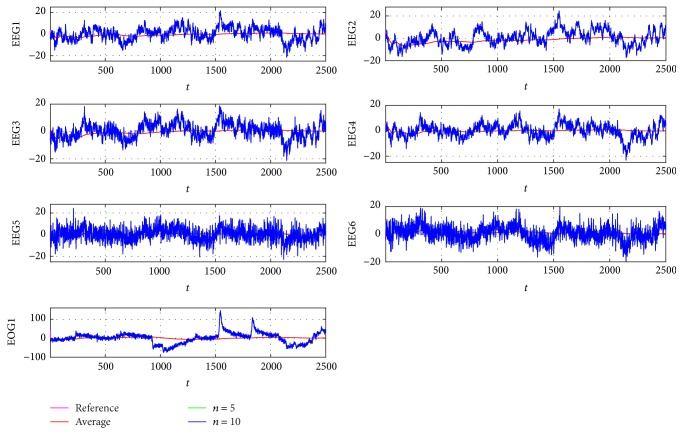
Identification for signals from Figure 13, comparing the sampled signal, the average approximation, and the identification by using 5 and 10 iterations with EFF.

As a second part of the test, the estimation-identification process is applied to sampled signals taken from [7, 23], which are for subject 1 from 6 Electroencephalogram (EEG) and one Electrooculogram (EOG) channels for different activities. The objective is to apply the EFF iterative description from case (c) to obtain the parameters that allow the approximation by using (2). The reference data as in Figure 13 have 10 seconds of recording with a sampling frequency of 250 Hz, obtaining in total 2500 samples representing instants *t*. The results of applying the average description and 5 and 10 iterations of the EFF are shown in Figure 14.

**Figure 15:**
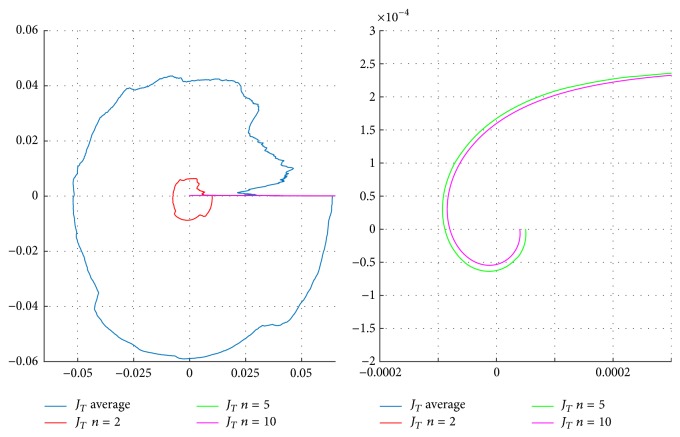
Polar representation of the combination of error functionals obtained by identifying the signal from Figure 14.

In Figure 14, signals from Figure 13 are separated to improve the convergence to each. These are different from one another. Having different signals is useful to determine the fact that the identification using the EFF is adequate for chaotic nonstationary cases, such as the EEG or EOG, presented in this work. To conclude, the measured error is viewed as the functional errors average from the seven signals for each estimation as shown in (13), where *n* is the number of iterations. Results are given in a polar graph in Figure 15, observing functionals errors that tend to zero in all corrected approximations, having a better performance when more iterations are applied.

(13) $$\begin{array}{c} {J_{T}}_{n}=\frac{J_{EEG1_{n}}+J_{EEG2_{n}}+\cdots +J_{EEG6_{n}}+J_{EOG1_{n}}}{7}. \end{array}$$

### 3.2. Classification of EEG Signals

In the previous section, the EEG signals parameter estimation was possible. Then we present the application of the estimated parameters into the classification of EEG signals viewed in our case as a stochastic system with multioutput EEG responses. For different instances, we consider the same database used in Section 3.1 [7, 23] and the estimation-identification process with iterative EFF iterating 10 times.

**Figure 16:**
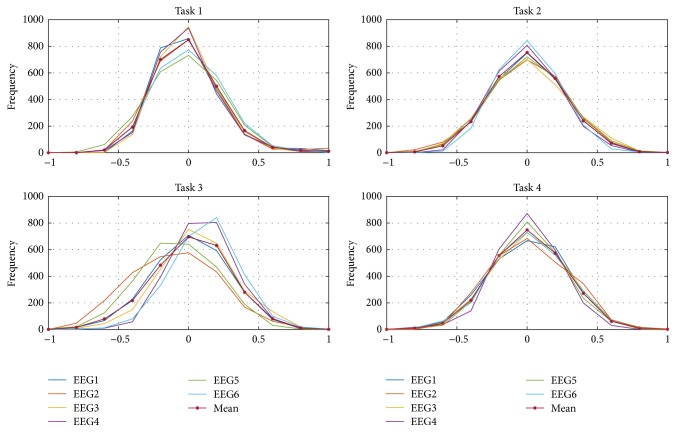
Four tasks defined by six EEG signals distributions with their representative mean distributions.

The regarded four tasks are multiplication (Task 1), letter (Task 2), rotation (Task 3). and counting (Task 4). For each task, six EEG channels (1 to 6) are considered having specific distributions as seen in Figure 16, which presents the normalized signals distributions divided into ten principal intervals between [−1, 1], for a better appreciation. The average of the six distributions signals for every task was obtained and presented also in Figure 16. The mean distribution is the representative one to be used as the base for the stochastic EEG classification.

**Figure 17:**
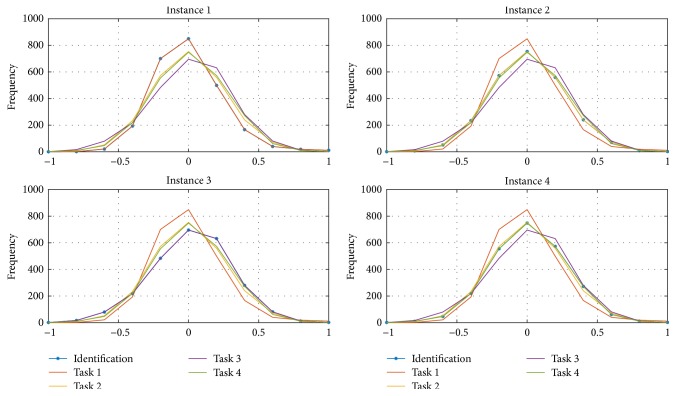
Comparison between the base EEG signal distributions and four identified signals instances.

For a specific task, when identifying its six signals, their corresponding distributions could be obtained as well as the mean identified representative distribution. The classification is made by comparing the four base distributions from Figure 16 to the mean identified and determining the convergence error between them. The assignment is then for the task where the minimum error is found.  Figure 17 presents four different instances.

**Figure 18:**
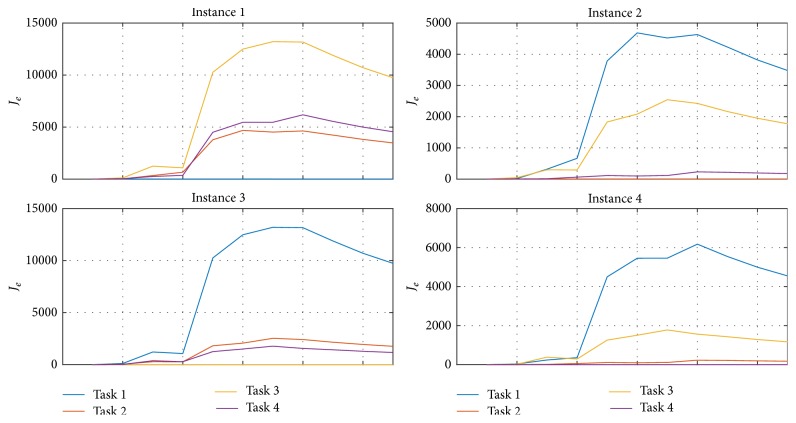
Functional errors comparing the EEG base and identified distributions from Figure 17.

In Figure 17 the similarities between the identification of different tasks are seen. For example, within instance 1, the identified distribution is closer to Task 1 than to the others, so that it is possible to say that the identification corresponds to it. To quantify how close they are, the recursive error functional [21] based on the second probability moment considering the errors from the distribution comparison is calculated and presented in Figure 18 for each instance.

From Figure 18, the minimum cumulative error corresponds to the correct assigned classification. On the other hand, Table 1 represents a decision chart based on the errors for the four instances, summarizing the classification process to minimum error identification, obtaining good results in all considered instances.

## 4. Conclusion

The results obtained for cases (a) and (b) are obvious because the changes are made considering the past, and the EFF description is for actual information. Therefore, the use of previous parameters would break the convergence after using the parameter obtained with the EFF leading to a poorer convergence.

For case (c) it could be said that special care must be taken when the time is important to obtain the identification because having more iterations, and in consequence less error, implies more execution time. However, the latency is big enough to allow a considerable number of iterations, modifying the EFF, and then the parameter as in case (c), from iteration 7 (as shown in Figures 7 and 8), results in better correction than that obtained by modifying only the parameters.

The estimation-identification process is adequate for nonlinear signals, such as those obtained from EEG. The importance of describing these signals lies in the description of possible missing information when drastic changes in concavity and frequency are given. Even when in this paper only time-variant analysis is shown, the reconstruction of chaotic signals gave good results in comparison with a simple average approach, as seen in Figure 14.

Even when the main objective of this work is the parameter estimation, a simple classification test has been performed to demonstrate one possible use of the parameters obtained by using our technique, achieving good results is the four presented instances (Table 1).

As future work, comparisons using more real signals should be performed, and finally, the obtained parameters could be helpful to create a database and obtain more characteristics to create useful synthetic signals and prove the effectiveness of new methods or techniques.

## Figures and Tables

**Table 1:** Classification of different instances of EEG signals considering the minimum error viewed as a decision chart from the cumulative errors.

Instance	Task 1	Task 2	Task 3	Task 4	Minimum error	Classification
1	**001.10**	485.26	772.26	525.93	001.10	Task 1
2	485.26	**001.43**	326.76	105.10	001.43	Task 2
3	772.26	325.10	**0.4333**	252.10	0.4333	Task 3
4	525.93	105.10	252.76	**0.4333**	0.4333	Task 4
